# Snail modulates JNK-mediated cell death in *Drosophila*

**DOI:** 10.1038/s41419-019-2135-7

**Published:** 2019-11-26

**Authors:** Chenxi Wu, Zhuojie Li, Xiang Ding, Xiaowei Guo, Ying Sun, Xingjun Wang, Yujia Hu, Tongtong Li, Xiaojin La, Jianing Li, Ji-an Li, Wenzhe Li, Lei Xue

**Affiliations:** 1 Institute of Intervention Vessel, Shanghai 10th People’s Hospital, Shanghai Key Laboratory of Signaling and Disease Research, School of Life Sciences and Technology, Tongji University, 1239 Siping Road, Shanghai, 200092 China; 2 College of Traditional Chinese Medicine, North China University of Science and Technology, 21 Bohai Road, Tangshan, 063210 China; 3 Center of Intervention Radiology, Zhuhai People’s Hospital, Zhuhai, 519000 China; 4 Present Address: Department of Neuroscience, Scripps Research Institute, 130 Scripps Way, Jupiter, Fl 33458 USA; 5 Present Address: Life Sciences Institute, Department of Cell and Developmental Biology, University of Michigan, Ann Arbor, MI 48109 USA

**Keywords:** Apoptosis, Developmental biology

## Abstract

Cell death plays a pivotal role in animal development and tissue homeostasis. Dysregulation of this process is associated with a wide variety of human diseases, including developmental and immunological disorders, neurodegenerative diseases and tumors. While the fundamental role of JNK pathway in cell death has been extensively studied, its down-stream regulators and the underlying mechanisms remain largely elusive. From a *Drosophila* genetic screen, we identified Snail (Sna), a Zinc-finger transcription factor, as a novel modulator of ectopic Egr-induced JNK-mediated cell death. In addition, *sna* is essential for the physiological function of JNK signaling in development. Our genetic epistasis data suggest that Sna acts downstream of JNK to promote cell death. Mechanistically, JNK signaling triggers dFoxO-dependent transcriptional activation of *sna*. Thus, our findings not only reveal a novel function and the underlying mechanism of Sna in modulating JNK-mediated cell death, but also provide a potential drug target and therapeutic strategies for JNK signaling-related diseases.

## Introduction

The Sna superfamily of transcription factors has been implicated in a broad spectrum of important biological functions, including mesoderm formation, epithelial–mesenchymal transition (EMT), tumor recurrence, immune regulation, neural differentiation, left–right identity, cell fate, and survival decisions^1–4^. Most of the Sna family members share a similar organization, with a evolutionarily conserved C-terminal domain that contains four–six C_2_H_2_-type Zinc-fingers for DNA binding, whereas the N terminus with a SNAG (Snail/Gfi) domain harbors the repressor activity^1^. As the first member of the Sna family, *sna* was identified as a critical regulator of mesoderm development in *Drosophila melanogaster*^5–7^. Although lacking the SNAG domain, *Drosophila* Sna has a consensus Pro-X-Asp-Leu-Ser-X-Lys (P-DLS-K) motif and executes its repressive function via interacting with a co-repressor, carboxy-terminal-binding protein (CtBP)^8,9^. As such, the fruit fly offers opportunities to investigate the physiological functions of Sna during development.

The c-Jun N-terminal Kinase (JNK) signaling is evolutionarily conserved from fruit fly to human, and plays crucial roles in regulating a wide range of cellular activities including proliferation, differentiation and migration, especially cell death^10,11^. This pathway can be triggered by various extrinsic and intrinsic signals, and is mediated through a mitogen-activated protein kinase (MAPK) cascade^12^. In *Drosophila*, the tumor necrosis factor (TNF) ortholog Eiger (Egr) binds to its receptor Grindelwald (Grnd), which in turn activates the conserved JNK cascade including dTAK1 (JNKK kinase), Hemipterous (Hep, the JNK kinase) and Basket (Bsk, the *Drosophila* JNK)^13–17^. Upon activation, Bsk phosphorylates and activates downstream transcription factors including the forkhead box O (FoxO), which modulates UV-induced Bsk-mediated cell death by directly up-regulating the pro-apoptotic gene *hid* expression^18,19^. Although tremendous effort has been made to complete the regulatory network centered on Egr-Bsk pathway in cell death^20–24^, the down-stream regulators and the underlying mechanisms remain poorly understood.

In this study, we used *Drosophila melanogaster* as an excellent in vivo system and identified Sna as a novel modulator of JNK pathway. First, our genetic analysis indicates that Sna functions downstream of JNK to regulate ectopically activated JNK-induced cell death in eye and wing development. We further show that loss of *sna* can block physiologically activated JNK signaling-induced cell death, and that Sna is necessary and sufficient for JNK-induced *puc* expression. Moreover, we demonstrate that Sna modulates dFoxO-triggered cell death. Finally, we provide evidence that gain of JNK signaling promotes dFoxO-dependent *sna* transcription. In conclusion, these findings reveal a previously unrecognized function of Sna in JNK signaling-mediated cell death, in addition to its well-accepted roles in development and EMT.

## Results

### Depletion of sna suppresses ectopic Egr-induced cell death in development

**Fig. 1:**
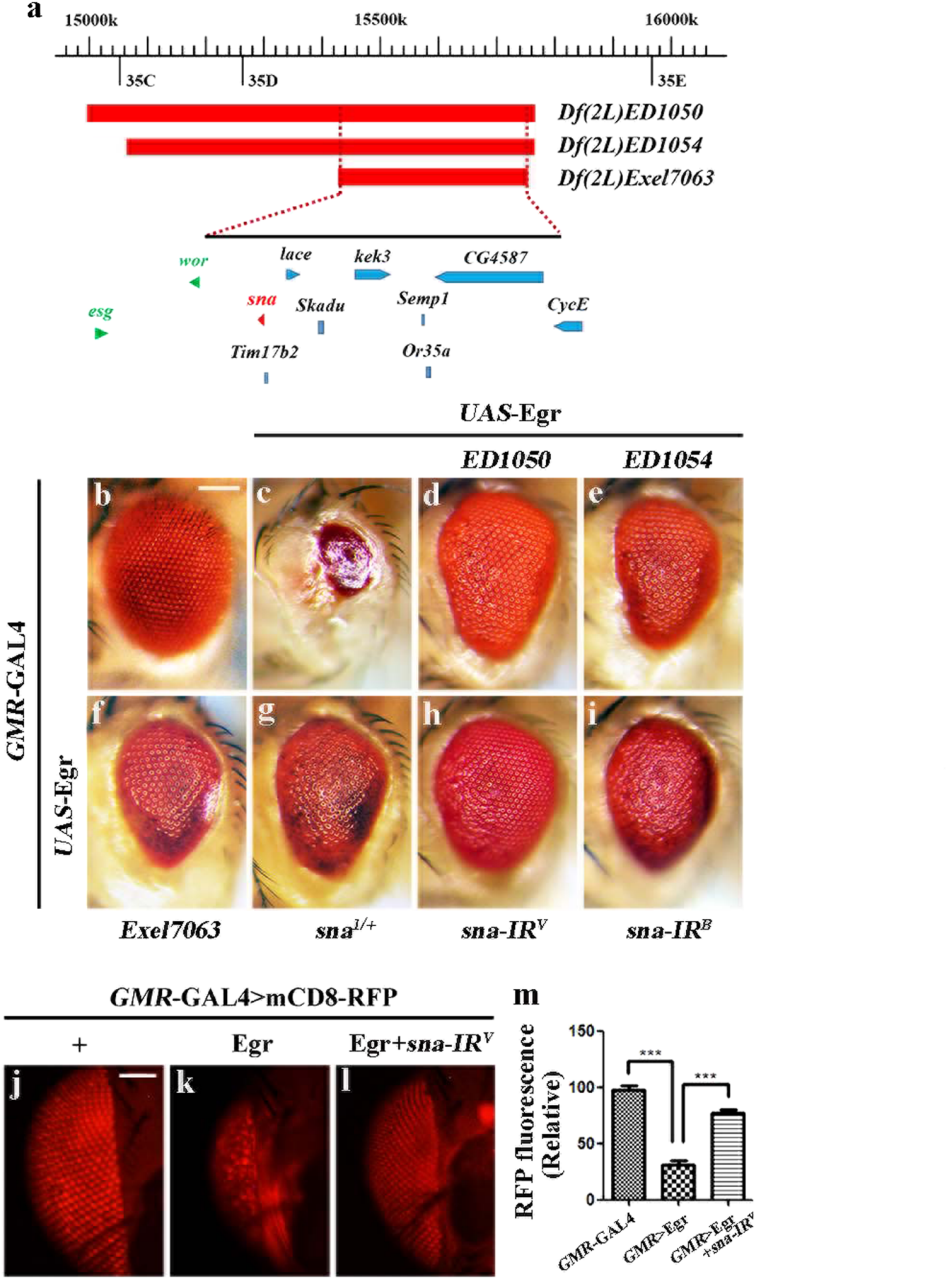
A genetic screen for dominant modifiers of *GMR* > Egr-induced eye-ablation phenotype. **a** A schematic depiction of the genomic region surrounding the *sna* locus. The three deficiencies *Df(2L)ED1050*, *Df(2L)ED1054*, and *Df(2L)Exel7063* are indicated. **b**–**i** Light micrographs of *Drosophila* adult eyes are shown. Compared with *GMR*-GAL4 control **b**, the *GMR* > Egr small eye phenotype **c** is considerably suppressed by deficiency *Df(2L)ED1050* **d**, *Df(2L)ED1054* **e**, or *Df(2L)Exel7063* **f** that deletes genes including *sna*, or in heterozygous *sna* mutant **g**, or by expressing two independent *sna* RNAi **h** and **i**. The sample size of *Drosophila* adult eye is 50. **j**–**l** Representative fluorescent microscopy images of adult eyes are shown. **m** Statistical analysis of fluorescence signals (*n* = 10) shown in figures **j**–**l**. Error bars indicates standard deviation. One-way ANOVA with Bonferroni multiple comparison test was used to compute *P*-values, ^***^P < 0.001. See the electronic supplementary material for detailed genotypes. Scale bar: 100 μm in **b**–**i**, 50 μm in **j**–**l**.

**Fig. 2:**
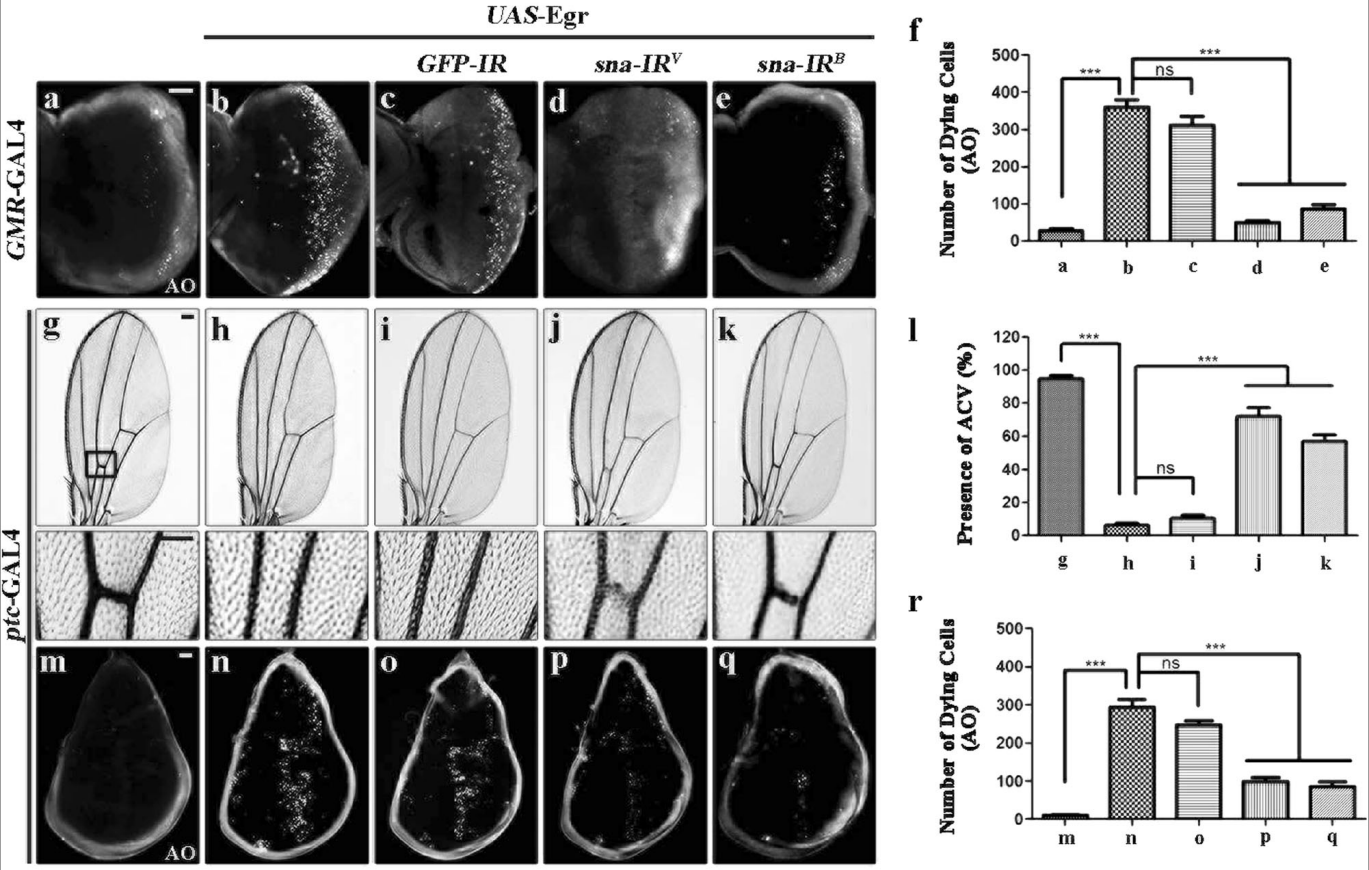
Depletion of *sna* suppresses Egr-triggered cell death in development. **a**–**e** Fluorescence micrographs of third instar larval eye discs are shown. Compared with the controls **a**, *GMR* > Egr-induced cell death in eye discs **b** remains unaffected by expression of a *GFP* RNAi **c**, but is dramatically impeded by knocking down *sna* **d** and **e**. **f** Statistical analysis of cell death in eye discs (*n* = 10) shown in figures **a**–**e**. Light micrographs of *Drosophila* adult wings **g**–**k** and fluorescence micrographs of third instar larval wing discs **m**–**q** are shown. Compared with the controls **g** and **m**, ectopic expression of Egr driven by *ptc*-GAL4 generates a loss-of-ACV phenotype in adults **h** and cell death in larval wing discs **n**, which are strongly blocked by RNAi-mediated depletion of *sna* **j**, **k**, **p** and **q**, but not that of *GFP* **i** and **o**. The lower panels show high magnification view of the boxed areas in upper panels **g**–**k**. Statistical analysis of the ACV phenotype in figures **g**–**k** (**l**, *n* = 20 for each genotype) and cell death in wing discs in figures **m**–**q** (**r**, *n* = 10) are shown. Error bars indicates standard deviation. One-way ANOVA with Bonferroni multiple comparison test was used to compute *P*-values, ***P < 0.001; ns, no significant difference. In all wings, anterior is to the left and distal up. *UAS-GFP-IR* is included **c**, **i**, **o** as a negative control to demonstrate that the suppressive effect of *UAS*-*sna-IR* is specific, but not a result of GAL4 titration by another UAS line. See the electronic supplementary material for detailed genotypes. Scale bars: 50 μm in **a**–**e, g**–**k** (lower panels) and **m**–**q**, 100 μm in **g**–**k** (upper panels).

Ectopic expression of the TNF ortholog Egr in *Drosophila* eyes driven by *GMR*-GAL4 (*GMR* > Egr) produces a small eye phenotype in the adult stage (Fig. 1b, c)^14,15^. and triggers apoptotic cell death posterior to the morphogenetic furrow (MF) in third instar eye discs, as revealed by acridine orange (AO) staining that detects dying cells (Fig. 2a, b)^25^, and anti-CDcp-1 antibody staining that specifically recognizes the cleaved effector caspase Dcp-1 (Supplementary Fig. 1a, b)^26^. As the fly eye is also the most accepted organ of the nervous system^27^, to quantify the extent of Egr-induced neuronal loss, we employed the *UAS*-mCD8-RFP (a fusion protein between mouse lymphocyte marker CD8 and the fluorescence protein) reporter system^28^, and found that ectopic Egr resulted in remarkable loss of the photoreceptor neurons in *Drosophila* adult eyes (Fig. 1j, k, m).

To identify additional factors that regulate Egr-induced cell death, we performed a genetic screen for dominant modifiers of the *GMR* > Egr-induced small eye phenotype using the Bloomington *Drosophila* Stock Center Deficiency kit^21–24,29^. One of the suppressors was mapped cytologically within 35D2-35D4, a region uncovered by three overlapping deficiencies *Df(2L)ED1050*, *Df(2L)ED1054*, and *Df(2L)Exel7063* (Fig. 1a). Amalgamating such deficiency into *GMR* > Egr background significantly suppressed the reduced eye size (Fig. 1d–f). This region contains nine genes including *snail* (*sna*) (Fig. 1a), the *Drosophila* ortholog of Sna superfamily^1^, which encodes a C_2_H_2_ zinc finger transcription factor involved in embryonic mesoderm development, EMT and asymmetric cell division^5,30–34^. Intriguingly, the *GMR* > Egr eye phenotype was suppressed to a similar extent in heterozygous *sna* mutants (Fig. 1g), suggesting loss of *sna* is responsible for the suppressive effect of the deficiencies. Consistently, *GMR* > Egr triggered small eye phenotype, cell death, and photoreceptor loss were notably inhibited by two independent *sna* RNA interference (RNAi) that target distinct regions of the *sna* transcript (Fig. 1h, i, l, m; 2d–f; Supplementary Fig. 1d–f). A quantitative reverse transcription polymerase chain reaction (qRT-PCR) assay was performed to verify the knockdown efficiencies of the two *sna* RNAi lines (Supplementary Fig. 2a). A *GFP* RNAi was employed as a negative control (Fig. 2c; Supplementary Fig. 1c). Collectively, these results indicate that the transcription factor Sna plays an essential role in ectopic Egr-triggered cell death during eye development.

In *Drosophila*, *sna*, *esgcargot* (*esg*) and *worniu* (*wor*) are considered to be functionally redundant Snail superfamily members that encode zinc finger transcription factors^1,35,36^. Although *esg* and *wor* are located in the vicinity of *sna* on the chromosome, they are not included in the region uncovered by *Df(2L)Exel7063* (Fig. 1a). We found that *GMR* > Egr-induced small eye phenotype was not visibly suppressed by depletion of *esg* or *wor* (Supplementary Fig. 2e–j), suggesting that *esg* and *wor* are not involved in Egr-induced cell death.

To examine whether Sna plays a more general role in cell death, we expressed the pro-apoptotic gene *head involution defective* (*hid*) by the *GMR*-GAL4 driver^37^, and found that *GMR* > Hid-induced small eye phenotype was not suppressed by knockdown of *sna* (Supplementary Fig. 2b–d), suggesting Sna is specifically involved in Egr-triggered cell death.

*Drosophila* adult wing represents another excellent model system to investigate cell death in development. Consistent with previous reports, expression of Egr along the anterior/posterior (A/P) compartment boundary driven by *patched* (*ptc*)-GAL4 also resulted in apoptotic cell death, revealed by AO staining (Fig. 2c, n) and anti-Cleaved Caspase-3 (CC-3) antibody staining (Supplementary Fig. 1g, h), a read-out of the initiator caspase (Caspase-9-like) DRONC activity^38^, and generated a loss of anterior cross vein (ACV) phenotype (Fig. 2g, h)^29,39^, which was phenocopied by expressing the cell death gene *grim*^23^. These phenotypes were considerably impeded by RNAi-mediated inactivation of *sna* (Fig. 2j–l, p–r; Supplementary Fig. 1j–l), but remained unaffected by expression of *GFP-IR* (Fig. 2i, o; Supplementary Fig. 1i), suggesting that Sna modulates ectopic Egr-promoted cell death in a non-tissue-specific manner.

### Sna acts downstream of Bsk to modulate cell death

**Fig. 3:**
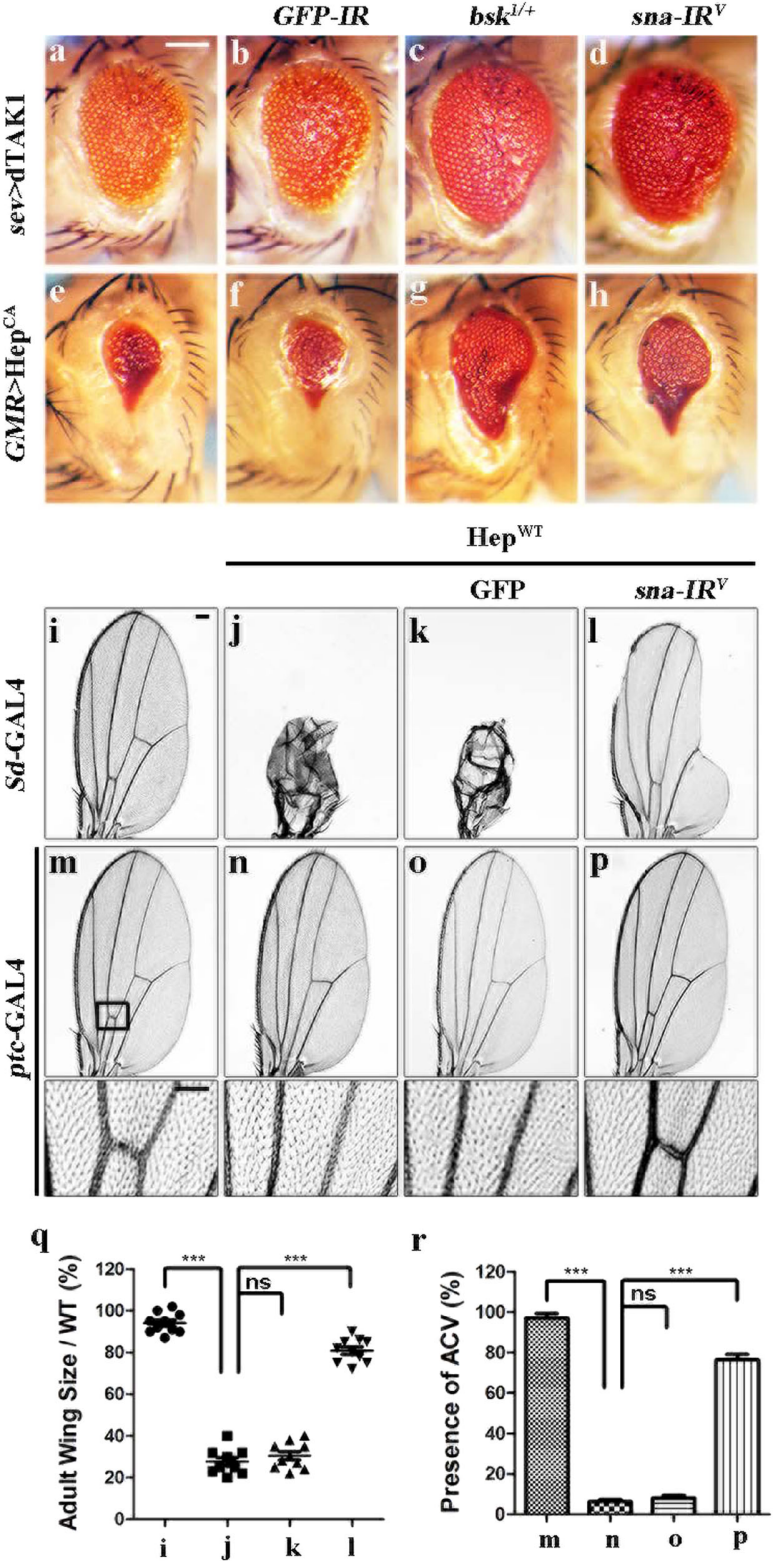
Sna acts down-stream of Hep in JNK-mediated cell death. **a**–**h** Light micrographs showing *Drosophila* adult eyes. The small and rough eye phenotype resulted from ectopic expression of dTAK1 **a** or Hep^CA^ **e** is evidently suppressed by mutating one copy of endogenous *bsk* **c** and **g**, or knocking-down *sna* **d** and **h**, but not *GFP* **b** and **f**, which served as a negative control. **i**–**p** Light micrographs of *Drosophila* adult wings. Compared with the controls **i** and **m**, the wing phenotypes of *Sd* > Hep^WT^ **j** and *ptc* > Hep^WT^ **n** flies are suppressed by expressing a *sna-IR* **l** and **p**, but not GFP **k** and **o**. In **m**–**p**, the lower panels are high magnification of the boxed areas in upper panels. Statistical analysis of the adult wing size/wild type (WT) **q** (*n* = 10) and the ACV phenotype **r** (*n* = 20 for each genotype) as shown in figures **i**–**l** and **m**–**p**, respectively. One-way ANOVA with Bonferroni multiple comparison test was used to compute *P*-values, ***P < 0.001; ns, no significant difference. In all wings, anterior is to the left and distal up. See the electronic supplementary material for detailed genotypes. Scale bars: 100 μm in **a**–**l** and **m**–**p** (upper panels**)**, 50 μm in **m**–**p** (lower panels).

Egr triggers both JNK-dependent and JNK-independent cell death in development^14,15,21^. To determine whether Sna is required for JNK-mediated cell death, we overexpressed dTAK1 (JNKKK) or Hep (JNKK) in the developing eyes. Eye-specific expression of dTAK1 driven by *sevenless* (*sev*)-GAL4 or a constitutive activated form of Hep (Hep^CA^) driven by *GMR*-GAL4 promoted extensive cell death in third instar larval eye discs, which were visualized by terminal deoxynucleotidyl transferase dUTP nick-end labeling (TUNEL) staining that labels both apoptotic and necrotic cells (Supplementary Fig. 3a, f)^40,41^, and generated rough eyes with reduced size (Fig. 3a, e)^29,42^. These phenotypes were suppressed by knockdown of *sna* (Fig. 3d, h; Supplementary Fig. 3d, e, I, j), but not that of *GFP* (Fig. 3b, f; Supplementary Fig. 3b, g), while heterozygous *bsk*^*1*^ mutants were included as a positive control (Fig. 3c, g; Supplementary Fig. 3c, h). In addition, expression of Puc, a phosphatase that negatively regulates Bsk activity^43^, near fully suppressed the small eye phenotype produced by *GMR* > Hep^CA^ (Supplementary Fig. 4). Together, *sna* is indispensable for Egr-induced Bsk-mediated cell death in eye development.

To investigate whether Sna modulates Bsk-mediated cell death in other cellular contexts, we activated Bsk signaling in the wing or dorsal thorax. Ectopic expression of wild type Hep in the wing pouch driven by *Scalloped* (*Sd*)-GAL4 (*Sd* > Hep^WT^) promotes broad-scale cell death^23^ that results in severely reduced adult wing blade (Fig. 3i, j), which was significantly suppressed by depleting *sna* (Fig. 3l, q). Accordantly, *ptc* > Hep^WT^-induced apoptosis in third larval wing discs (Supplementary Fig. 3k, l) and loss-of-ACV phenotype in adult wings (Fig. 3m, n) were inhibited by expressing a *sna* RNAi, but not GFP (Fig. 3o, p, r; Supplementary Fig. 3m–o). In addition, we found that expression of Hep driven by *pnr*-Gal4 (*pnr* > Hep^WT^) triggers cell death in the thorax^22^ and produces a small scutellum phenotype, which is partially suppressed by depleting *sna*, but not *GFP* (Supplementary Fig. 5). Hence, we conclude that Sna regulates JNK-mediated cell death down-stream of Hep in a non-tissue-specific manner.

Moreover, ectopic expression of Bsk under the control of *GMR*-GAL4 generates a small and rough eye phenotype (Supplementary Fig. 6a, b), which is suppressed by knockdown of *sna* (Supplementary Fig. 6d), but not expression of GFP (Supplementary Fig. 6c), suggesting that Sna acts down-stream of the JNK cascade to modulate cell death. In addition, ectopic expression of Sna is sufficient to trigger a large scale of cell death in the eye and wing discs (Supplementary Fig. 6e–i, n, s–u), and produce a small eye phenotype in the adulthood (Supplementary Fig. 6j). Consistent with the notion that Sna acts downstream of Bsk, the *GMR* > Sna-induced cell death and eye phenotype could not be suppressed by expressing Bsk^DN^, while *sna-IR* and LacZ served as positive and negative controls, respectively (Supplementary Fig. 6k–m, o–r).

### *sna* is required for the physiological functions of Bsk

**Fig. 4:**
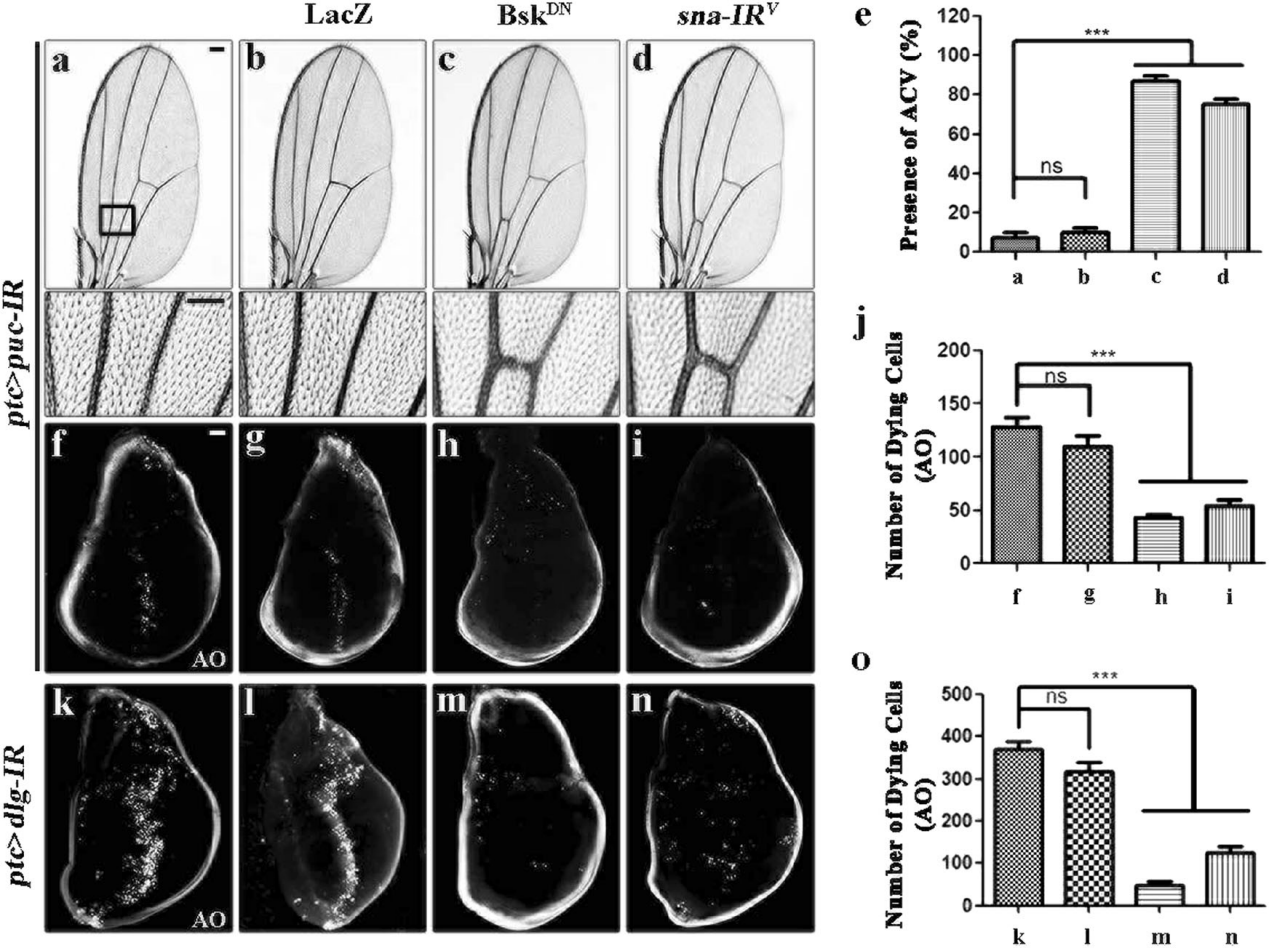
*sna* is required for the physiological functions of Bsk. Light micrographs of *Drosophila* adult wings **a**–**d** and fluorescence micrographs of third instar larval wing discs **f**–**i** and **k**–**n** are shown. RNAi-mediated down-regulation of *puc* along the A/P compartment boundary by *ptc*-GAL4 triggers the loss-of-ACV phenotype in adult wings **a**, which is resulted from cell death in larval wing discs **f**. Both phenotypes depend on endogenous Bsk **c** and **h** and Sna **d** and **i**. The bottom panels show high magnification views of the boxed areas in upper panels **a**–**d**. Knockdown of *dlg* along the A/P compartment boundary (*ptc* > *dlg-IR*) induces Bsk-dependent cell death **k** and **m**, which is significantly blocked by depletion of *sna* **n**. LacZ expression is used as a negative control **b**, **g**, and **l**. **e**, **j**, and **o** Statistical analysis of ACV phenotype (*n* = 20 for each genotype) and cell death in wing discs (*n* = 10) as shown in figures **a**–**d**, **f**–**i**, and **k**–**n**, respectively. Error bars indicates standard deviation. One-way ANOVA with Bonferroni multiple comparison test was used to compute *P*-values, ***P < 0.001; ns, no significant difference. In all wings, anterior is to the left and distal up. See the electronic supplementary material for detailed genotypes. Scale bars: 100 μm in **a**–**d** (upper panels), 50 μm in **a**–**d** (lower panels) and **f**–**n**.

The above data imply that Sna acts as a crucial factor down-stream of Bsk to modulate cell death, yet it remains unknown whether Sna is involved in the physiological role of JNK signaling. To address this question, we knocked down *puc*, which encodes a JNK phosphatase that negatively regulates JNK activity^43^, along the A/P compartment boundary of wing discs by *ptc*-GAL4 (*ptc* > *puc-IR*), and observed strong cell death accompanied with increased caspase activity in third-instar larval wing discs (Fig. 4f; Supplementary Fig. 7a), and loss-of-ACV in adult wings (Fig. 4a). All the phenotypes, resulted from the activation of endogenous JNK signaling, were blocked by knockdown of *sna*, while expression of a dominant negative Bsk (Bsk^DN^) or LacZ served as a positive or negative control, respectively (Fig. 4bb–e, g–j; Supplementary Fig. 7b–d, i).

It has been reported that loss of cell polarity promotes JNK-mediated cell death in development^44–46^. In agreement with this view, knockdown of the cell polarity gene *disc large* (*dlg*) along the A/P compartment boundary by *ptc*-GAL4 (*ptc* > *dlg-IR*) induces severe cell death (Fig. 4k) and up-regulated caspase activity (Supplementary Fig. 7e) in third instar larval wing discs. Both phenotypes were appreciably blocked by depletion of *sna* or expression of Bsk^DN^, but remained unaffected by the expression of LacZ (Fig. 4l–o; Supplementary Fig. 7f–i). Thus, these data indicate that Sna contributes to the physiological function of Bsk signaling in regulating stress-induced cell death in development.

### Sna mediates FoxO-triggered cell death

**Fig. 5:**
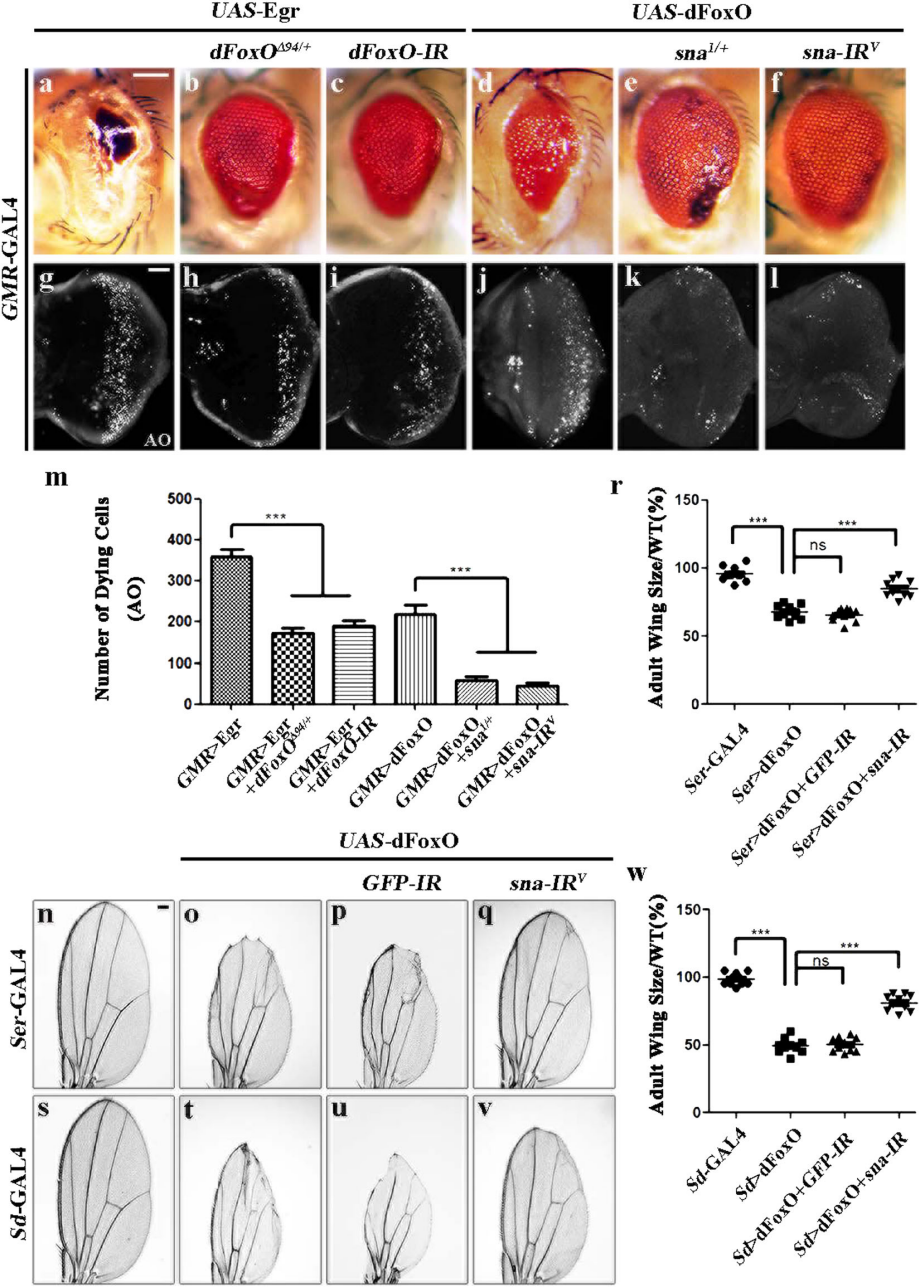
Sna regulates FoxO-mediated cell death. Light micrographs of *Drosophila* adult eyes **a**–**f**, adult wings **n**–**q**, and **s**–**v** and fluorescence micrographs of third instar larval eye discs **g**–**l** are shown. The small eye phenotype and massive cell death, produced by *GMR* > Egr **a** and **g**, are obviously suppressed in heterozygous *dFoxO*^*Δ94*^ background **b** and **h** or by knockdown of *dFoxO* **c** and **i**. Expression of dFoxO driven by *GMR*-GAL4 results in reduced eye size and increased cell death in eye discs **d** and **j**, which are suppressed by mutating one copy of endogenous *sna* **e** and **k** or RNAi-mediated depletion of *sna* **f** and **l**. Compared with the controls **n** and **s**, the wing phenotypes of *Ser* > dFoxO **o** and *Sd* > dFoxO **t** flies are partially suppressed by depletion of *sna* **q** and **v**, but not that of *GFP* **p** and **u**. In all wings, anterior is to the left and distal up. Statistical analysis of AO staining **m**, the adult wing size/wild type (WT) **r** and **w** as shown in figures **g**–**l**, **n**–**q**, and **s**–**v**, respectively (*n* = 10). One-way ANOVA with Bonferroni multiple comparison test was used to compute *P*-values, ***P < 0.001; ns, no significant difference. See the electronic supplementary material for detailed genotypes. Scale bar: 100 μm in **a**–**f**, **n**–**q** and **s**–**v**, 50 μm in **g**–**l**.

The transcription factor forkhead box O (FoxO) is a known down-stream regulator of Bsk-mediated cell death in response to stress^18,19^. Consistently, we found that *GMR* > Egr-induced cell death in eye discs and small eye phenotype were significantly impeded in heterozygous *dFoxO*^*Δ94*^ mutants, or by RNAi-mediated knockdown of *dFoxO* (Fig. 5a–c, g–i, m), suggesting dFoxO also regulates Egr-triggered Bsk-mediated cell death. To investigate the mechanism by which Sna regulates JNK-mediated cell death, we examined the genetic interaction between *sna* and *dFoxO*. Over-expression of dFoxO driven by *GMR*-GAL4 triggers intensive cell death in third instar eye discs and produces adult eyes with reduced size (Fig. 5d, j)^18,44^. We found that both phenotypes were dramatically suppressed in heterozygous *sna* mutants (Fig. 5e, k), and near fully blocked by RNAi-mediated depletion of *sna* (Fig. 5f, l). Moreover, we found dFoxO expression in wing development driven by *ptc*-GAL4, *Serrate* (*Ser*)-GAL4, or *Scalloped* (*Sd*)-GAL4 triggered extensive cell death and generated various wing defects in their corresponding areas, which are efficiently blocked by RNAi-mediated depletion of *sna*, but not that of *GFP* (Fig. 5n–w; Supplementary Fig. 8). Taking these data together, we conclude that Sna is required for FoxO-induced cell death in eye and wing development.

*Scutoid* (*Sco*) was originally identified as a dominant mutation resulted from a chromosomal transposition that affects *sna*, and is regarded as a *sna* gain-of-function allele^45^. Intriguingly, we found that *GMR* > dFoxO-induced small eye phenotype is notably enhanced in heterozygous *Sco* mutants (Supplementary Fig. 9), confirming that gain of Sna exacerbates FoxO-induced cell death.

### JNK signaling activates *sna* transcription

**Fig. 6:**
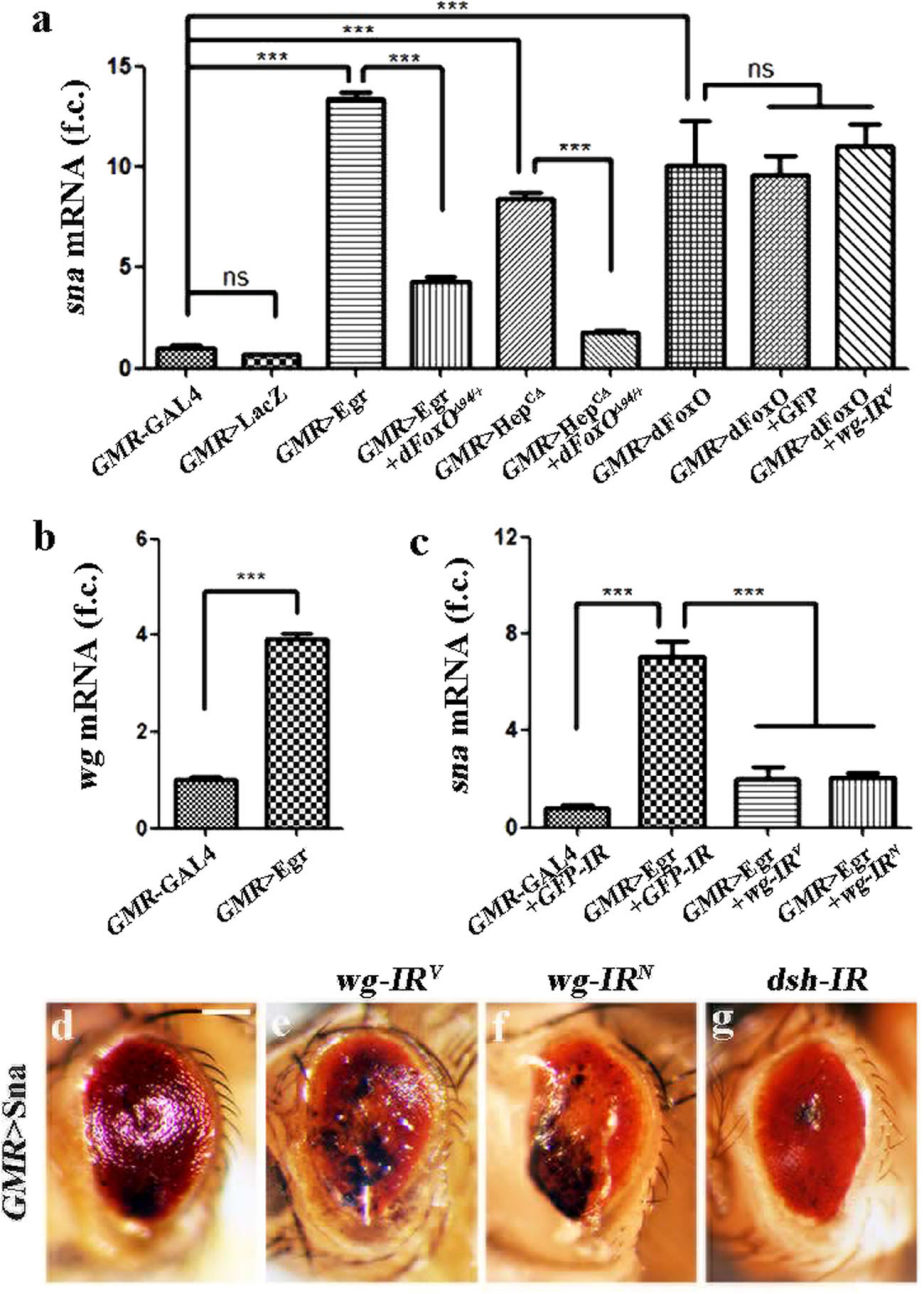
JNK signaling activates *sna* transcription. Histogram showing the levels of *sna* **a** and **c** and *wg* **b** mRNAs as measured by qRT-PCR. Error bars represent standard deviation from three independent experiments. One-way ANOVA with Bonferroni multiple comparison test or unpaired two-tailed *t*-test was used to compute *P*-values, ***P < 0.001. Light micrographs of *Drosophila* adult eyes **d**–**g**. The small eye phenotype produced by *GMR* > Sna **d** is not suppressed by RNAi-mediated depletion of *wg* or *dsh* **e**–**g**. See the electronic supplementary material for detailed genotypes. Scale bar: 100 μm in **d**–**g**.

The above data suggest that Sna is necessary and sufficient for Bsk-FoxO-signaling-induced cell death in development. Since FoxO encodes a transcription factor, we hypothesized that JNK signaling may activate *sna* transcription in a FoxO-dependent manner. To test this, we activated JNK signaling in the eye by ectopic expression of Egr or Hep, and checked *sna* mRNA level by the qRT-PCR assay. In support of our assumption, endogenous *sna* transcription was evidently up-regulated by ectopic Egr or Hep, and this activation was significantly blocked in heterozygous *dFoxO*^*Δ94*^ mutants (Fig. 6a), suggesting that JNK-induced *sna* expression depends on dFoxO. Consistently, the level of *sna* mRNA was also dramatically up-regulated by ectopic expression of dFoxO, but remained unaffected by that of LacZ (Fig. 6a). Thus, we conclude that JNK signaling triggers FoxO-dependent transcriptional activation of *sna*, which is necessary and sufficient for JNK-mediated cell death in development.

*wingless* (*wg*) is a recognized target of JNK pathway^24,46,47^ and elicits expression of Sna family transcription factors in the peripheral fly eye^48^. To further dissect the function of Wg in Egr/JNK/FoxO/Sna axis, we first performed the qRT-PCR assay to check *wg* mRNA level in *GMR* > Egr eyes. In line with the previous studies, we found that ectopic Egr is sufficient to activate *wg* transcription (Fig. 6b). Next, we checked whether Wg is required for JNK-mediated *sna* expression. To this end, we employed two independent *wg* RNAi and noticed a strong suppressive effects on the elevation of *sna* mRNA level triggered by Egr (Fig. 6c), but not that induced by FoxO (Fig. 6a), suggesting that Wg may contribute to Egr-induced *sna* transcription in parallel with FoxO. However, the *GMR* > Sna-induced small eye phenotype could not be blocked by knockdown of *wg* or *disheveled* (*dsh*) (Fig. 6d–g), which encodes a scaffold protein as the Wg transducer^49^. Thus, we confirmed that *wg* is involved in Egr/JNK-mediated *sna* transcription, but not required for Sna-promoted cell death.

### Sna induces puc activation in vivo

**Fig. 7:**
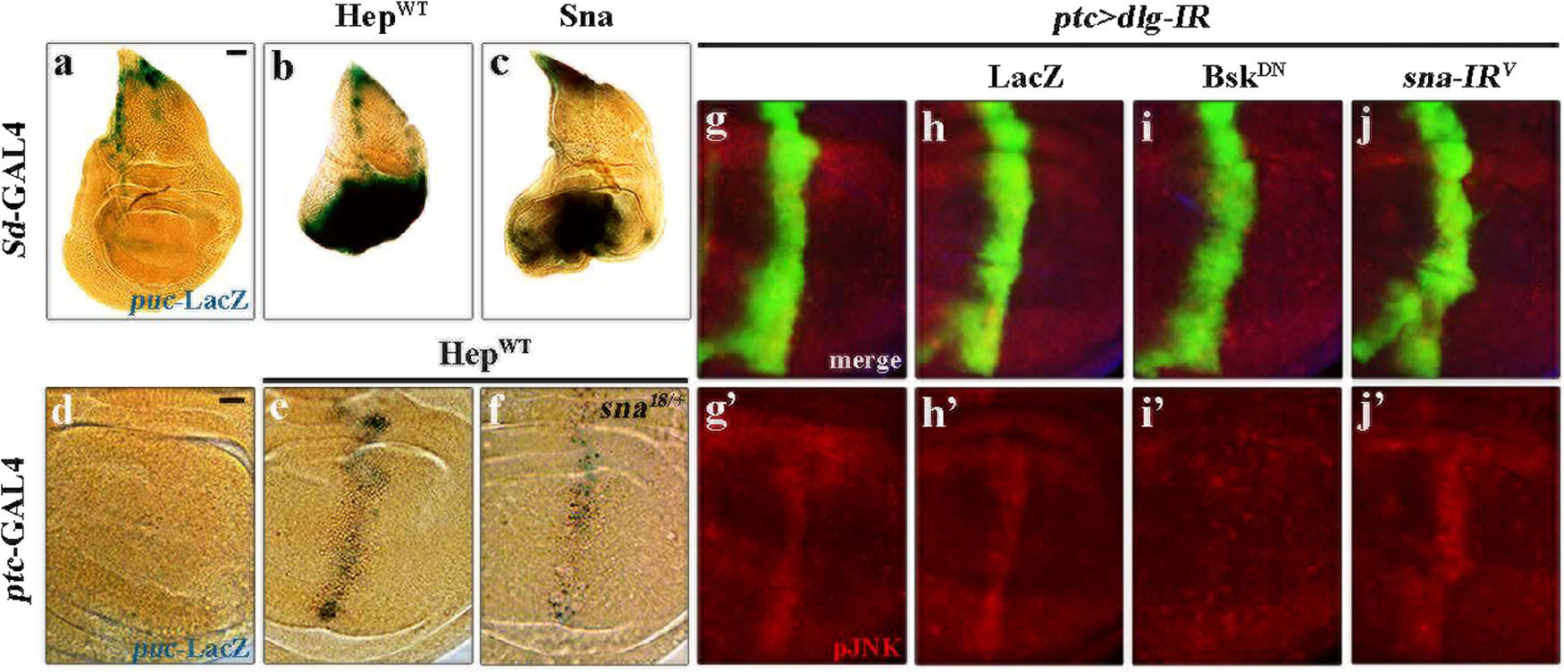
*sna* activates *puc* transcription in vivo. **a**–**f** Light micrographs showing X-Gal staining of a *puc*-LacZ reporter in wing discs. Compared with the control **a**, overexpression of Hep^WT^ or Sna in the wing pouch notably upregulated *puc* transcription **b** and **c**. Compared with *ptc*-GAL4 **d**, the increased *puc*-LacZ expression triggered by Hep^WT^ along the A/P compartment boundary **e** is partially impeded in heterozygous *sna* mutant **f**. **g**–**j** Fluorescence micrographs of *Drosophila* third instar larval wing discs are shown. The elevated JNK phosphorylation induced by knockdown of *dlg* **g** and **g**′ remains unaffected by RNAi-mediated depletion of *sna* **j** and **j**′. LacZ and Bsk^DN^ expressions are used here as negative and positive controls, respectively **h**, **h**′, **i** and **i**′. See the electronic supplementary material for detailed genotypes. Scale bar: 50 μm in **a**–**c**, 20 µm in **d**–**j**.

The above data suggest that Sna is a crucial downstream factor mediating JNK-dependent cell death. Next, to check if Sna could activate *puc* transcription in vivo, we examined the expression of *puc*-LacZ reporter by executing an X-Gal-staining assay^42^. Compared with the *Sd*-GAL4 control, expression of Hep^WT^ or Sna strongly induces up-regulation of *puc*-LacZ (Fig. 7a–c). Furthermore, the activation of *puc* in wing pouch along the A/P boundary triggered by *ptc* > Hep^WT^ could be moderately impeded by mutation in *sna* (Fig. 7d–f), suggesting Sna is both necessary and sufficient for JNK-mediated *puc* expression. Consistent with the genetic data that Sna acts downstream of Bsk, depletion-of-*dlg*-triggered JNK phosphorylation was inhibited by expression of Bsk^DN^, but not that of a *sna* RNAi or LacZ (Fig. 7g–j).

## Discussion

With the sophisticated genetic tools and conserved cell death machinery, *Drosophila* has been widely considered as an excellent model organism to unravel novel regulators of the cell death program during the last two decades^50–52^. In this study, we took advantage of the fly genetics and identified Sna as a novel regulator of Egr-induced cell death from a deficiency screen. Our genetic epistasis analysis established Sna as a crucial downstream mediator of the Egr-JNK-FoxO signaling in cell death. Mechanistically, Egr-JNK pathway activates *sna* transcription via FoxO. Previous work has reported that Wg signaling elicits expression of Sna family transcription factors in eye peripheral retinal apoptosis^48^. In addition, Zhang et al. reported that *wg* is required for *GMR* > Egr-induced cell death, and that *wg* expression is activated by Jun/Fos-mediated Egr-JNK signaling^24^. Thus, we are curious if Wg is required for JNK-mediated *sna* transcription. Our data demonstrate that Wg also contributes to Egr-induced *sna* transcription, but is not involved in the FoxO-promoted *sna* expression. These data suggest that the Egr-JNK signaling activates *sna* transcription by at least two independent mechanisms: FoxO and Jun/Fos-Wg. Moreover, we found Sna is sufficient to activate *puc* transcription, implying JNK activates *puc* expression via multiple means. Intriguingly, different from the previously reported functional redundancy in development^1,36^, the other two *Drosophila* Sna family proteins Esg and Wor are not involved in Egr-induced JNK-mediated cell death. Given the evolutionary conserved role of Sna and JNK, the data presented in this study may suggest a novel function of mammalian Sna family proteins in JNK-mediated cell death, in addition to its well-accepted roles in EMT, cell fate decision and survival.

## Materials and methods

### Fly strains

Flies were kept on a cornmeal and agar medium at 25 °C according to standard protocols. *Drosophila* strains used include: *sna*^*1*^ (25127), *sna*^*18*^ (3299), *UAS-sna-IR*^*B*^ (28679), *UAS*-mGFP (32197), *bsk*^*1*^ (3088), *UAS-*Bsk, *UAS*-dFoxO (9575), *Ser*-GAL4 (6791), *Sco*/*CyO* (2555), *UAS*-mCD8-RFP (32219), *UAS-dsh-IR* (31306), *wor*^*1*^(3155), *wor*^*4*^ (25170) and the deficiency kit were obtained from Bloomington *Drosophila* stock center. *UAS-sna-IR*^*V*^ (6263), *UAS-puc-IR* (3018), *UAS*-*dlg-IR* (41136), and *UAS-wg-IR*^*V*^ (13352) were obtained from Vienna *Drosophila* RNAi center. *UAS-wg-IR*^*N*^ (4889R-4), *UAS-esg-IR*^*N-1*^ (3758R-1), and *UAS-esg-IR*^*N-2*^ (3758R-5) were received from Fly Stocks of National Institute of Genetics (NIG-FLY). *UAS-GFP-IR* (0355) and *UAS-wor-IR* (GL00186) were obtained from TsingHua Fly Center. *GMR*-GAL4^53^; *Sd*-GAL4 and *ptc*-GAL4^39^*; UAS*-Egr, *UAS*-Egr^W^, and *UAS*-Hid^14^; *UAS*-Bsk^DN^, *UAS*-Puc, and *puc*^*E69*^^42^*; sev*-GAL4, *UAS*-dTAK1, *UAS*-Hep^CA^, *UAS*-Hep^WT^, *UAS*-LacZ, *pnr-*GAL4, and *UAS*-GFP^22,29,54^; *dFoxO*^*Δ94*^ and *UAS-dFoxO-IR*^39^ were previously described. *UAS*-Sna^74*b*^ fly was a kind gift of J. Kumar. For all fly cross experiments, healthy unmated male and female parents were randomly assigned to different groups. Double-blinded method was employed during the experiments.

### AO staining

Eye and wing discs were dissected from third instar larvae in PBST and incubated in 1 × 10^−5^ M AO for 5 min at room temperature prior to imaging as described^21^.

### TUNEL staining

The wing and eye discs were dissected from wandering third-instar larvae in PBS. Discs were fixed in 4% paraformaldehyde for 30 min at room temperature and washed with PBS-Tx (0.3% Triton100) three times for 30 min. TUNEL staining was performed using the Fluorescein Cell Death Kit produced by Boster Company.

### X-Gal staining

Wing discs were dissected from third instar larvae in PBST (1 × PBS pH 7.0, 0.1% Triton X-100) and stained for ß-galactosidase activity as described^21^.

### qRT-PCR

TRIzol (Invitrogen) was used to isolate total RNA from 10 wing imaginal discs dissected from third instar larvae or 30 adult heads collected from freshly eclosed flies of indicated genotypes, and qRT-PCR was performed as previously described^55^ using following primers:

For *rp49* Sense: 5′-TACAGGCCCAAGATCGTGAA-3′

Antisense: 5′-TCTCCTTGCGCTTCTTGGA-3′

For *sna* Sense: 5′-ATGGCCGCCAACTACAAAAG-3′

Antisense: 5′-GCAAACTGTGAGTCCTTGGTC-3′

For *wg* Sense: 5′-CCAAGTCGAGGGCAAACAGAA-3′

Antisense: 5′-TGGATCGCTGGGTCCATGTA-3′.

### Immunohistochemistry

Imaginal wing discs dissected from third instar larvae were collected in cold PBS and fixed in 4% paraformaldehyde. After proper washes, the wing discs were blocked in 10% horse serum, and stained with antibodies. The following antibodies were used: rabbit anti-Cleaved Dcp-1 (1:100, Cell Signaling Technology, Cat. #9578), rabbit anti-Cleaved Caspase-3 (1:200, Cell Signaling Technology, Cat. #9661), and rabbit anti-phospho-JNK (1:200, Calbiochem, Cat. #559309). Secondary antibody was goat anti-Rabbit-Cyanine3 (1:1000, Life technologies, Cat. #A10520). Vectashield mounting media (Vector Laboratories, Cat. #H-1500) was used for mounting.

### Data and statistics

All data were verified in at least three independent experiments. Results are presented as bar graphs or scatter plots created using GraphPad Prism 6.0. A combination of unpaired two-tailed *t*-test and one-way ANOVA with Bonferroni’s multiple comparison test was used to calculate statistical significance. Center values’ as mean. Error bars indicates standard deviation. *P* value < 0.05 was considered significant. ns is not significant, *P* ≥ 0.05; * is *P* < 0.05; ** is *P* < 0.01; *** is *P* < 0.001. *P* values are included in the relevant figure legends.

## Supplementary information

Supplementary Figure 1

Supplementary Figure 2

Supplementary Figure 3

Supplementary Figure 4

Supplementary Figure 5

Supplementary Figure 6

Supplementary Figure 7

Supplementary Figure 8

Supplementary Figure 9

Supplementary Figure Legends
