# Supplementary material for: Snail modulates JNK-mediated cell death in Drosophila
Source: Cell Death Dis. 2019 Nov 26;10(12):893. doi: 10.1038/s41419-019-2135-7 (PMC6879600; doi:10.1038/s41419-019-2135-7)
Supplement: Supplementary file 10 — Supplementary Figure Legends [file 41419_2019_2135_MOESM10_ESM.docx]

**Snail modulates JNK-mediated cell death in *Drosophila***

Chenxi Wu^1,2,#^, Zhuojie Li^1,#^, Xiang Ding^1^, Xiaowei Guo^1^, Ying Sun^1^, Xingjun Wang^1,4^, Yujia Hu^1,5^, Tongtong Li^2^, Xiaojin La^2^, Jianing Li^2^, Ji-an Li^2^, Wenzhe Li^1^ and Lei Xue^1,3*^

^1^ Institute of Intervention Vessel, Shanghai 10th People's Hospital, Shanghai Key Laboratory of Signaling and Disease Research, School of Life Science and Technology, Tongji University, 1239 Siping Road, Shanghai 200092, China

^2^ College of Traditional Chinese Medicine, North China University of Science and Technology, 21 Bohai Road, Tangshan 063210, China

^3^ Center of Intervention Radiology, Zhuhai People's Hospital, Zhuhai 519000, China

^4^ Present Address: Department of Neuroscience, Scripps Research Institute, 130 Scripps Way Jupiter, Florida 33458, USA

^5^ Present Address: Life Sciences Institute, Department of Cell and Developmental Biology, University of Michigan, Ann Arbor, MI 48109, USA

^#^ These authors contribute equally to this work

^*^ Correspondence: [lei.xue@tongji.edu.cn](mailto:lei.xue@tongji.edu.cn)

**Supplementary Information**

**Supplementary Figure Legends**

**Detailed Genotypes**

**Supplementary Figure Legends**

**Figure S1. Depletion of *sna* suppresses Egr-triggered cell death in development.**

Fluorescence micrographs of 3^rd^ instar larval eye discs stained with anti-Cleaved Dcp-1 (Asp216) antibody (CDcp-1) (**a**-**e**) or wing discs stained with anti-Cleaved Caspase-3 (CC-3) antibody (**g**-**k**). Compared with the controls (**a** and **g**), overexpressing Egr posterior to the MF by *GMR*-Gal4 (**b**) or along the A/P boundary by *ptc*-gal4 (**h**) results in increased apoptotic cell death and caspase activity in the corresponding areas, both of which are suppressed by knocking-down *sna* (**d**, **e**, **j** and **k**). *GFP-IR* is used here as a negative control (**c** and **i**). Quantifications of apoptotic cell death by CDcp-1 (**f**) and CC-3 antibody (**l**) (n=10) staining as shown in figures **a**-**e** and **g**-**k**, respectively. Error bars indicates standard deviation. One-way ANOVA with Bonferroni multiple comparison test was used to compute *P*-values, significance is indicated with asterisks (****P*<0.001). ns stands for not significant. Scale bars: 50μm in **a**-**e** and **g**-**k**.

Genotypes: (**a**) *GMR*-GAL4/+, (**b**) *UAS*-Egr^R^/+; *GMR*-GAL4/+, (**c**) *UAS*-Egr^R^/+; *GMR*-GAL4/*UAS-GFP-IR*, (**d**) *UAS*-Egr^R^/+; *GMR*-GAL4/*UAS*-*sna-IR^V^*, (**e**) *UAS*-Egr^R^/+; *GMR*-GAL4/*UAS*-*sna-IR^B^*, (**g**) *ptc*-GAL4/+, (**h**) *ptc*-GAL4/*UAS*-Egr^W^, (**i**) *ptc*-GAL4/*UAS*-Egr^W^; *UAS-GFP-IR*/+, (**j**) *ptc*-GAL4/*UAS*-Egr^W^; *UAS*-*sna-IR^V^*/+ and (**k**) *ptc*-GAL4/*UAS*-Egr^W^; *UAS*-*sna-IR^B^*/+.

**Figure S2. The roles of Sna family members in cell death.**

(**a**) The knock-down efficacies of *sna* RNAi lines. Expression of two independent *sna* RNAi significantly reduces the level of *sna* mRNA, as measured by quantitative RT-PCR. Total RNA of *Drosophila* third instar larvae wing discs (n=10, in each group) are extracted and normalized for cDNA synthesis. Error bars represents standard deviation from three independent experiments. One-way ANOVA with Bonferroni multiple comparison test was used to compute *P*-values, ^**^*P*<0.01, ^***^*P*<0.001. (**b**-**j**) Light micrographs of *Drosophila* adult eyes are shown. The *GMR*-Hid small eye phenotype (**b**) remains unaffected by expressing two independent *sna* RNAi (**c** and **d**). Compared with the GFP control (**e**), the *GMR*>Egr small eye phenotype is not suppressed by mutating one copy of endogenous *wor* (**h** and **i**), or by depletion of *esg* (**f** and **g**) or *wor* (**j**). Scale bar: 100μm in **b**-**j**.

Genotypes: (**a**) (Control) *Sd*-GAL4/+, (*sna-IR^V^*) *Sd*-GAL4/+; *UAS-sna-IR^V^*/+, (*sna-IR^B^*) *Sd*-GAL4/+; *UAS-sna-IR^B^*/+, (**b**) *GMR*-Hid/+, (**c**) *GMR*-Hid/*UAS-sna-IR^V^*, (**d**) *GMR*-Hid/*UAS-sna-IR^B^*, (**e**) *UAS*-Egr^R^/+; *GMR*-GAL4/*UAS*-GFP, (**f**) *UAS*-Egr^R^/+; *GMR*-GAL4/*UAS-esg-IR^N-1^*, (**g**) *UAS*-Egr^R^/+; *GMR*-GAL4/*UAS-esg-IR^N-2^*, (**h**) *UAS*-Egr^R^/*wor^1^*; *GMR*-GAL4/*+*, (**i**) *UAS*-Egr^R^/*wor^4^*; *GMR*-GAL4/*+* and (**j**) *UAS*-Egr^R^/+; *GMR*-GAL4/*UAS-wor-IR*.

oi**Figure S3. Sna acts down-stream of Hep in JNK-mediated cell death.**

Fluorescence micrographs of third instar larval discs stained with TUNEL (**a**-**d** and **f**-**i**) or CC-3 antibody (**k**-**n**). The massive apoptosis triggered by *sev*>dTAK1 (**a**) and *GMR*>Hep^CA^ (**f**), which visualized by TUNEL staining, are evidently suppressed by mutating one copy of endogenous *bsk* (**c** and **h**), or knockdown of *sna* (**d** and **i**), but not *GFP* (**b** and **g**). Compared with the *ptc*-GAL4 (**k**), the increased caspase activity in wing discs of *ptc>*Hep^WT^ (**l**) is suppressed by knockdown of *sna* (**n**), but not GFP (**m**). Quantifications of apoptotic cell death stained by TUNEL (**e** and **j**) and CC-3 antibody (**o**) (n=10) staining as shown in figures **a**-**d, f**-**i** and **k**-**n**, respectively. Error bars indicates standard deviation. One-way ANOVA with Bonferroni multiple comparison test was used to compute *P*-values, significance is indicated with asterisks (****P*<0.001). ns stands for not significant. Scale bars: 50μm in **a**-**d, f**-**i** and **k**-**n**.

Genotypes: (**a**) *sev*-GAL4 *UAS*-dTAK1/+, (**b**) *sev*-GAL4 *UAS*-dTAK1/+; *UAS-GFP-IR*/+, (**c**) *sev*-GAL4 *UAS*-dTAK1/*bsk^1^*, (**d**) *sev*-GAL4 *UAS*-dTAK1/+; *UAS*-*sna-IR^V^*/+, (**f**) *GMR-*GAL4 *UAS*-Hep^CA^*/*+, (**g**) *GMR-*GAL4 *UAS*-Hep^CA^/*UAS-GFP-IR*, (**h**) *bsk^1^*/+; *GMR-*GAL4 *UAS*-Hep^CA^*/*+, (**i**) *GMR-*GAL4 *UAS*-Hep^CA^/*UAS*-*sna-IR^V^*, (**k**) *ptc*-GAL4/+, (**l**) *ptc*-GAL4/*UAS*-Hep^WT^, (**m**) *ptc*-GAL4/*UAS*-Hep^WT^; *UAS*-GFP/+ and (**n**) *ptc*-GAL4/*UAS*-Hep^WT^; *UAS*-*sna-IR^V^*/+.

**Figure S4. Puc inhibites *GMR*>Hep^CA^-induced small eye phenotype.**

Light micrographs showing *Drosophila* adult eyes. The small eye phenotype triggered by *GMR*>Hep^CA^ (**a**) is blocked by overexpressing Puc (**b**). Scale bar: 100μm in **a**-**b**.

Genotypes: (**a**) *GMR-*GAL4 *UAS*-Hep^CA^*/*+ and (**b**) *GMR-*GAL4 *UAS*-Hep^CA^*/UAS*-Puc.

**Figure S5. Loss of *sna* suppresses Hep-triggered cell death in thorax development.**

Light micrographs showing *Drosophila* adult notum. Compared with the control (**a**), the small scutellum phenotype of *pnr*>Hep^WT^ (**b**) is partially impeded by RNAi-mediated knocking-down of *sna* (**d**), but not that of *GFP* (**c**). Scale bar: 100μm in **a**-**d**.

Genotypes: (**a**) *pnr*-GAL4/+, (**b**) *UAS*-Hep^WT^/+; *pnr*-GAL4/+, (**c**) *UAS*-Hep^WT^/+; *pnr*-GAL4/*UAS*-*GFP-IR* and (**d**) *UAS*-Hep^WT^/+; *pnr*-GAL4/*UAS*-*sna-IR^V^*.

**Figure S6. *sna* acts downstream of Bsk to modulate cell death.**

Light micrographs of *Drosophila* adult eyes (**a**-**d** and **j**-**m**) and fluorescence micrographs of third instar larval eye (**e**, **f** and **n**-**q**) and wing (**g**, **h**, **s** and **t**) discs are shown. Compared with the control (**a**), the rough eye phenotype produced by *GMR*>Bsk (**b**) is suppressed by expressing a *sna RNAi* (**d**), but not GFP (**c**). Compared with the controls (**e**, **g** and **s**), ectopic expression of Sna driven by *GMR*- or *ptc*-GAL4 triggers cell death detected by AO or CC-3 antibody staining in the eye (**f**) or wing disc (**h** and **t**). The small eye phenotype and apoptosis in 3^rd^ instar larval eye discs triggered by *GMR*>Sna (**j** and **n**) are suppressed by expressing a *sna RNAi* (**l** and **p**), but not LacZ (**k** and **o**) or Bsk^DN^ (**m** and **q**). Statistical analysis of cell death stained by AO (**i**), TUNEL (**r**) and CC-3 antibody (**u**) (n=10) as shown in figures **e**-**h**, **n**-**q**, **s** and **t**, respectively. Error bars indicates standard deviation. One-way ANOVA with Bonferroni multiple comparison test or unpaired two-tailed t-test was used to compute *P*-values, ^***^*P*<0.001; ns, no significant difference. Scale bar: 100μm in **a**-**d** and **j**-**m**, 50μm in **e**-**h**, **n**-**q**, **s** and **t**.

Genotypes: (**a**) *GMR*-GAL4/+, (**b**) *GMR*-GAL4/*UAS*-Bsk, (**c**) *GMR*-GAL4/*UAS*-Bsk; *UAS*-GFP/+, (**d**) *GMR*-GAL4/*UAS*-Bsk; *UAS*-*sna-IR^V^*/+, (**e**) *GMR*-GAL4/+, (**f**) *UAS*-Sna^74b^/+; *GMR*-GAL4/+, (**g** and **s**) *ptc*-GAL4/+, (**h** and **t**) *ptc*-GAL4/*UAS*-Sna^74b^, (**j** and **n**) *UAS*-Sna^74b^/+; *GMR*-GAL4/+, (**k** and **o**) *UAS*-Sna^74b^/+; *GMR*-GAL4/*UAS*-LacZ, (**l** and **p**) *UAS*-Sna^74b^/+; *GMR*-GAL4/*UAS*-*sna-IR^V^* and (**m** and **q**) *UAS*-Sna^74b^/+; *GMR*-GAL4/*UAS*-Bsk^DN^.

**Figure S7. *sna* is required for the physiological functions of Bsk.**

Fluorescence micrographs of third instar larval wing discs (**a-h**) are shown. Knockdown of *puc* or *dlg* by *ptc*-GAL4 triggers increased caspase activity in 3^rd^ instar larval wing discs (**a** and **e**). Both phenotypes depend on endogenous Bsk (**c** and **g**) and Sna (**d** and **h**). LacZ expression is used as a negative control (**b** and **f**). (**i**) Statistical analysis of CC-3 activity in wing discs (n=10) as shown in figures **a-h**. Error bars indicates standard deviation. One-way ANOVA with Bonferroni multiple comparison test was used to compute *P*-values, ^***^*P*<0.001; ns, no significant difference. Scale bars: 50μm in **a**-**h**.

Genotypes: (**a**) *ptc*-GAL4/*UAS*-*puc-IR*, (**b**) *ptc*-GAL4/*UAS*-*puc-IR*; *UAS-*LacZ/+, (**c**) *ptc*-GAL4/*UAS*-*puc-IR*; *UAS-*Bsk^DN^/+, (**d**) *ptc*-GAL4/*UAS*-*puc-IR*; *UAS*-*sna-IR^V^*/+, (**e**) *ptc*-GAL4/+; *UAS*-*dlg-IR*/+, (**f**) *ptc*-GAL4/+; *UAS*-*dlg-IR*/*UAS-*LacZ, (**g**) *ptc*-GAL4/+; *UAS*-*dlg-IR*/*UAS-*Bsk^DN^ and (**h**) *ptc*-GAL4/+; *UAS*-*dlg-IR*/*UAS*-*sna-IR^V^*.

**Figure S8. *sna* is required for *ptc*>dFoxO-induced phenotypes*.***

(**a**-**d**) Light micrographs showing *Drosophila* adult wings. Compared with the control (**a**), the wing phenotype of *ptc*>dFoxO (**b**) is partially suppressed by depletion of *sna* (**d**), but not that of *GFP* (**c**). In **a**-**d,** the lower panels are high magnification of the boxed areas in upper panels. In all wings, anterior is to the left and distal up. Fluorescence micrographs showing AO staining of 3^rd^ instar larval wing discs (**f**-**i** and **k**-**n**). Compared with the controls (**f** and **k**), the elevated cell death induced by *Ser>*dFoxO (**g**) or *Sd>*dFoxO (**l**) is partially suppressed by depletion of *sna* (**i** and **n**), but not that of *GFP* (**h** and **m**). Statistical analysis of the ACV phenotype (**e**) and AO staining (**j** and **o**) (n=10) as shown in figures **a**-**d**, **f**-**i** and **k**-**n**, respectively. One-way ANOVA with Bonferroni multiple comparison test was used to compute *P*-values, ^***^*P*<0.001; ns, no significant difference. Scale bars: 50μm in **a**-**d** (lower panels), **f**-**i** and **k**-**n**, 100μm in **a**-**d** (upper panels).

Genotypes: (**a**) *ptc*-GAL4/+, (**b**) *ptc*-GAL4/*UAS*-dFoxO, (**c**) *ptc*-GAL4/*UAS*-dFoxO; *UAS-GFP-IR*/+ and (**d**) *ptc*-GAL4/*UAS*-dFoxO; *UAS*-*sna-IR^V^*/+, (**f**) *Ser*-GAL4/+, (**g**) *Ser*-GAL4/*UAS*-dFoxO, (**h**) *Ser*-GAL4/*UAS*-dFoxO; *UAS-GFP-IR*/+, (**i**) *Ser*-GAL4/*UAS*-dFoxO; *UAS*-*sna-IR^V^*/+, (**k**) *Sd*-GAL4/+, (**l**) *Sd*-GAL4/+; *UAS*-dFoxO/+, (**m**) *Sd*-GAL4/+; *UAS*-dFoxO/+; *UAS-GFP-IR*/+ and (**n**) *Sd*-GAL4/+; *UAS*-dFoxO/+; *UAS*-*sna-IR^V^*/+.

**Figure S9. Gain-of-Sna aggravates dFoxO-induced cell death in eye development.**

Light micrographs showing *Drosophila* adult eyes. *Sco* mutation enhances *GMR*>dFoxO-induced small eye phenotype. Scale bar: 100μm in **a**-**c**.

Genotypes: (**a**) *GMR*-GAL4/*Sco*, (**b**) *GMR*-GAL4 *UAS*-dFoxO/+ and (**c**) *GMR*-GAL4 *UAS*-dFoxO/*Sco*.

**Detailed Genotypes**

**Figure 1**

(**b**) *GMR*-GAL4/+

(**c**) *UAS*-Egr^R^/+; *GMR*-GAL4/+

(**d**) *UAS*-Egr^R^/*Df(2L)ED1050*; *GMR*-GAL4/+

(**e**) *UAS*-Egr^R^/*Df(2L)ED1054*; *GMR*-GAL4/+

(**f**) *UAS*-Egr^R^/*Df(2L)Exel7063*; *GMR*-GAL4/+

(**g**) *UAS*-Egr^R^/*sna^1^*; *GMR*-GAL4/+

(**h**) *UAS*-Egr^R^/+; *GMR*-GAL4/*UAS*-*sna-IR^V^* (V6232)

(**i**) *UAS*-Egr^R^/+; *GMR*-GAL4/*UAS*-*sna-IR^B^* (BL28679)

(**j**) *UAS*-mCD8-RFP/+; *GMR*-GAL4/+

(**k**) *UAS*-Egr^R^/*UAS*-mCD8-RFP; *GMR*-GAL4/+

(**l**) *UAS*-Egr^R^/*UAS*-mCD8-RFP; *GMR*-GAL4/*UAS*-*sna-IR^V^* (V6232)

**Figure 2**

(**a**) *GMR*-GAL4/+

(**b**) *UAS*-Egr^R^/+; *GMR*-GAL4/+

(**c**) *UAS*-Egr^R^/+; *GMR*-GAL4/*UAS-GFP-IR*

(**d**) *UAS*-Egr^R^/+; *GMR*-GAL4/*UAS*-*sna-IR^V^* (V6232)

(**e**) *UAS*-Egr^R^/+; *GMR*-GAL4/*UAS*-*sna-IR^B^* (BL28679)

(**g** and **m**) *ptc*-GAL4/+

(**h** and **n**) *ptc*-GAL4/*UAS*-Egr^W^

(**i** and **o**) *ptc*-GAL4/*UAS*-Egr^W^; *UAS-GFP-IR*/+

(**j** and **p**) *ptc*-GAL4/*UAS*-Egr^W^; *UAS*-*sna-IR^V^*/+

(**k** and **q**) *ptc*-GAL4/*UAS*-Egr^W^; *UAS*-*sna-IR^B^*/+

**Figure 3**

(**a**) *sev*-GAL4 *UAS*-dTAK1/+

(**b**) *sev*-GAL4 *UAS*-dTAK1/+; *UAS-GFP-IR*/+

(**c**) *sev*-GAL4 *UAS*-dTAK1/*bsk^1^*

(**d**) *sev*-GAL4 *UAS*-dTAK1/+; *UAS*-*sna-IR^V^*/+

(**e**) *GMR-*GAL4 *UAS*-Hep^CA^*/*+

(**f**) *GMR-*GAL4 *UAS*-Hep^CA^/*UAS-GFP-IR*

(**g**) *bsk^1^*/+; *GMR-*GAL4 *UAS*-Hep^CA^*/*+

(**h**) *GMR-*GAL4 *UAS*-Hep^CA^/*UAS*-*sna-IR^V^*

(**i**) *Sd*-GAL4/+

(**j**) *Sd*-GAL4/+; *UAS*-Hep^WT^/+

(**k**) *Sd*-GAL4/+; *UAS*-Hep^WT^/+; *UAS*-GFP/+

(**l**) *Sd*-GAL4/+; *UAS*-Hep^WT^/+; *UAS*-*sna-IR^V^*/+

(**m**) *ptc*-GAL4/+

(**n**) *ptc*-GAL4/*UAS*-Hep^WT^

(**o**) *ptc*-GAL4/*UAS*-Hep^WT^; *UAS*-GFP/+

(**p**) *ptc*-GAL4/*UAS*-Hep^WT^; *UAS*-*sna-IR^V^*/+

**Figure 4**

(**a** and **f**) *ptc*-GAL4/*UAS*-*puc-IR*

(**b** and **g**) *ptc*-GAL4/*UAS*-*puc-IR*; *UAS-*LacZ/+

(**c** and **h**) *ptc*-GAL4/*UAS*-*puc-IR*; *UAS-*Bsk^DN^/+

(**d** and **i**) *ptc*-GAL4/*UAS*-*puc-IR*; *UAS*-*sna-IR^V^*/+

(**k**) *ptc*-GAL4/+; *UAS*-*dlg-IR*/+

(**l**) *ptc*-GAL4/+; *UAS*-*dlg-IR*/*UAS-*LacZ

(**m**) *ptc*-GAL4/+; *UAS*-*dlg-IR*/*UAS-*Bsk^DN^

(**n**) *ptc*-GAL4/+; *UAS*-*dlg-IR*/*UAS*-*sna-IR^V^*

**Figure 5**

(**a** and **g**) *UAS*-Egr^R^/+; *GMR*-GAL4/+

(**b** and **h**) *UAS*-Egr^R^/*+*; *GMR*-GAL4/*dFoxO^Δ94^*

(**c** and **i**) *UAS*-Egr^R^/*+*; *GMR*-GAL4/*UAS-dFoxO-IR*

(**d** and **j**) *GMR*-GAL4 *UAS*-dFoxO/+

(**e** and **k**) *GMR*-GAL4 *UAS*-dFoxO/*sna^1^*

(**f** and **l**) *GMR*-GAL4 *UAS*-dFoxO/+; *UAS*-*sna-IR^V^*/+

(**n**) *Ser*-GAL4/+

(**o**) *Ser*-GAL4/*UAS*-dFoxO

(**p**) *Ser*-GAL4/*UAS*-dFoxO; *UAS-GFP-IR*/+

(**q**) *Ser*-GAL4/*UAS*-dFoxO; *UAS*-*sna-IR^V^*/+

(**s**) *Sd*-GAL4/+

(**t**) *Sd*-GAL4/+; *UAS*-dFoxO/+

(**u**) *Sd*-GAL4/+; *UAS*-dFoxO/+; *UAS-GFP-IR*/+

(**v**) *Sd*-GAL4/+; *UAS*-dFoxO/+; *UAS*-*sna-IR^V^*/+

**Figure 6**

(**a**) From left to right: (1) *GMR*-GAL4/+, (2) *GMR*-GAL4/*UAS*-LacZ, (3) *UAS*-Egr^W^/+; *GMR*-GAL4/+, (4) *UAS*-Egr^W^/+; *GMR*-GAL4/*dFoxO^Δ94^*, (5) *GMR*-GAL4 *UAS*-Hep^CA^/+, (6) *GMR*-GAL4 *UAS*-Hep^CA^/*dFoxO^Δ94^*, (7) *UAS*-dFoxO/+; *GMR*-GAL4/+, (8) *UAS*-dFoxO/+; *GMR*-GAL4/*UAS*-GFP, (9) *UAS*-dFoxO/+; *GMR*-GAL4/*UAS-wg-IR^V^*

(**b**) From left to right: (1) *GMR*-GAL4/+, (2) *UAS*-Egr^R^/+; *GMR*-GAL4/+

(**c**) From left to right: (1) *GMR*-GAL4/*UAS-GFP-IR*, (2) *UAS*-Egr^R^/+; *GMR*-GAL4/*UAS-GFP-IR*, (3) *UAS*-Egr^R^/+; *GMR*-GAL4/*UAS-wg-IR^V^*, (4) *UAS*-Egr^R^/*UAS-wg-IR^N^*; *GMR*-GAL4/+

(**d**) *UAS*-Sna^74b^/+; *GMR*-GAL4/+

(**e**) *UAS*-Sna^74b^/+; *GMR*-GAL4/*UAS-wg-IR^V^*

(**f**) *UAS*-Sna^74b^/*UAS-wg-IR^N^*; *GMR*-GAL4/+

(**g**) *UAS*-Sna^74b^/+; *GMR*-GAL4/*UAS-dsh-IR*

**Figure 7**

(**a**) *Sd*-GAL4/+; *puc^E69^*/+

(**b**) *Sd*-GAL4/+; *UAS*-Hep^WT^/+; *puc^E69^*/+

(**c**) *Sd*-GAL4/+; *UAS*-Sna^74b^/+; *puc^E69^*/+

(**d**) *ptc*-GAL4/+; *puc^E69^*/+

(**e**) *ptc*-GAL4/*UAS*-Hep^WT^; *puc^E69^*/+

(**f**) *ptc*-GAL4 *UAS*-Hep^WT^/*sna^18^*; *puc^E69^*/+

(**g**) *ptc*-GAL4/+; *UAS*-*dlg-IR*/+

(**h**) *ptc*-GAL4/+; *UAS*-*dlg-IR*/*UAS-*LacZ

(**i**) *ptc*-GAL4/+; *UAS*-*dlg-IR*/*UAS-*Bsk^DN^

(**j**) *ptc*-GAL4/+; *UAS*-*dlg-IR*/*UAS*-*sna-IR^V^*
